# Lifetime body size and reproductive factors: comparisons of data recorded prospectively with self reports in middle age

**DOI:** 10.1186/1471-2288-11-7

**Published:** 2011-01-17

**Authors:** Benjamin J Cairns, Bette Liu, Suzanne Clennell, Rachel Cooper, Gillian K Reeves, Valerie Beral, Diana Kuh

**Affiliations:** 1 Cancer Epidemiology Unit, University of Oxford, Oxford, UK; 2 MRC Unit for Lifelong Health and Ageing, University College and Royal Free Medical School, London, UK; 3 National Centre in HIV Epidemiology and Clinical Research, Faculty of Medicine, University of New South Wales, Sydney, Australia

## Abstract

**Background:**

Data on lifetime exposures are often self-reported in epidemiologic studies, sometimes many years after the relevant age. Validity of self-reported data is usually inferred from their agreement with measured values, but few studies directly quantify the likely effects of reporting errors in body size and reproductive history variables on estimates of disease-exposure associations.

**Methods:**

The MRC National Survey of Health and Development (NSHD) and the Million Women Study (MWS) are UK population-based prospective cohorts. The NSHD recruited participants at birth in 1946 and has followed them at regular intervals since then, whereas the MWS recruited women in middle age. For 541 women who were participants in both studies, we used statistical measures of association and agreement to compare self-reported MWS data on body size throughout life and reproductive history, obtained in middle age, to NSHD data measured or reported close to the relevant ages. Likely attenuation of estimates of linear disease-exposure associations due to the combined effects of random and systematic errors was quantified using regression dilution ratios (RDRs).

**Results:**

Data from the two studies were very strongly correlated for current height, weight and body mass index, and age at menopause (Pearson r = 0.91-0.95), strongly correlated for birth weight, parental heights, current waist and hip circumferences and waist-to-height ratio (r = 0.67-0.80), and moderately correlated for age at menarche and waist-to-hip ratio (r = 0.52-0.57). Self-reported categorical body size and clothes size data for various ages were moderately to strongly associated with anthropometry collected at the relevant times (Spearman correlations 0.51-0.79). Overall agreement between the studies was also good for most quantitative variables, although all exhibited both random and systematic reporting error. RDRs ranged from 0.66 to 0.86 for most variables (slight to moderate attenuation), except weight and body mass index (1.02 and 1.04, respectively; little or no attenuation), and age at menarche, birth weight and waist-to-hip ratio (0.44, 0.59 and 0.50, respectively; substantial attenuation).

**Conclusions:**

This study provides some evidence that self-reported data on certain anthropometric and reproductive factors may be adequate for describing disease-exposure associations in large epidemiological studies, provided that the effects of reporting errors are quantified and the results are interpreted with caution.

## Background

Epidemiologic studies often use exposure information that is recalled or otherwise self-reported, and the suitability of such data for use in epidemiological analyses is commonly inferred from their agreement with measured values. A range of studies have found that self-reported data on anthropometry, clothes sizes and other body size variables are often valid in that they agree with measured values to within a reasonable accuracy [1-16]. However, there is consistent evidence across these studies of systematic errors in self-reports, including under-reporting of weight that is greater among heavier individuals. Women's reproductive history and related data, including whether they were breastfed, age at menarche, age at menopause and use of exogenous hormones, are also self-reported with reasonable accuracy [10,17-21] which may vary according to educational attainment [22]. For the purposes of many epidemiological studies, what matters most are the effects of random and systematic errors on estimates and interpretation of disease-exposure associations. But few studies have attempted to directly quantify the likely effects on epidemiological analyses of reporting errors in body size or reproductive history variables [23-27].

The Medical Research Council (MRC) National Survey of Health and Development (NSHD) is a prospective cohort study of a sample of men and women born in England, Scotland and Wales who were recruited at birth in March 1946 and have been followed regularly throughout life by physical measurement, nurse interview and questionnaire [28]. The Million Women Study (MWS) is a prospective cohort study of women, mainly born in 1934-1948 and recruited in middle age from England and Scotland, which uses postal questionnaires to obtain information on various exposures of interest including reproductive history and body size at different ages [29]. For women who were participants in both studies, we compared self-reported information from the MWS with corresponding NSHD data and examined how reporting errors could affect estimation of disease-exposure relationships.

## Methods

The MRC National Survey of Health and Development (NSHD) is a socially stratified birth cohort of 2,547 women and 2,815 men, followed since their births in a single week in March, 1946 [28]. Data have been collected by physical measurement, interview and questionnaire on a range of variables at intervals throughout life. With study members currently in their sixties, the purpose of the NSHD is now to investigate how lifetime experience and exposures affect healthy ageing http://www.nshd.mrc.ac.uk.

The Million Women Study (MWS) is a prospective cohort study of 1.3 million women recruited through National Health Service (NHS) Breast Screening Centres in England and Scotland during 1996 to 2001 [29]. At recruitment, women were asked about their current health and a range of other variables, and have been resurveyed every 3-4 years with further questions. Study questionnaires are available to view at http://www.millionwomenstudy.org.

Women participating in the MWS with dates of birth in the same week in March 1946 as the NSHD were matched by NHS number to female participants in the NSHD. In this validation study, self-reported MWS data on a range of body size, reproductive history and related variables were compared to NSHD data on the same or similar information, where the latter were measured or collected close to the relevant age (Table 1). For these analyses, all anthropometry was recorded in imperial units and was converted to metric units. Duration data recorded in numbers of months were converted to years by division by 12, without rounding. MWS data on ages at menarche and menopause were available only in whole numbers of years. Because the mean age of 12 year olds (for example) is 12 years and 6 months, 0.5 was added to each MWS age value to allow quantitative comparison with the more precise NSHD data on age at these events.

**Table 1:** Corresponding Million Women Study (MWS) and National Survey of Health and Development (NSHD) variable descriptions

		Age(s) when		Average age
	NSHD variables	recorded	MWS variables	of reporting
Variable*	(measured, M, or reported, R)	(years)	(self-reported)	(years)†
Birth weight	Birth weight (M)	0	Birth weight	55.3
Whether breastfed	Duration breastfed (R by mother)	2	Whether breastfed (yes/no)	55.3
Mother's height	Mother's height (R by parent)	6	Mother's height	55.3
Father's height	Father's height (R by parent)	6	Father's height	55.3
Body size, age 10	Body mass index (M)	11	Relative body size at age 10	55.3
Age at menarche	Whether periods started and age (R by mother)	14-15	Age at which periods started	52.1
Body size, age 20	Body mass index (R)	20	Clothes size at age 20	55.3
Use of oral contraceptives	Ever use of oral contraceptives (R)	31, 43	Ever use of oral contraceptives	52.1
Age at menopause	Date of last period if stopped for 12+ months (R)	47-54, 57	Age when periods stopped for 6+ months	52.1
Weight	Weight (M)	53	Weight	52.1
Height	Height (M)	53	Height	52.1
Body size	BMI derived from weight and height (M)	53	Clothes size at resurvey	55.3
Waist circumference	Waist circumference (M)	53	Waist circumference	55.3
Hip circumference	Hip circumference (M)	53	Hip circumference	55.3
Chest circumference	Chest circumference (M)	53	Bra band size at resurvey	55.3

* The following derived variables were also compared between the studies: Body mass index (derived from Weight and Height); Waist-to-hip ratio; Waist-to-height ratio.

† Among women matched to both studies.

All participants gave written informed consent to take part in each study, and approval for this validation study was provided by the Cambridgeshire 4 Research Ethics Committee (MWS) and the Central Manchester Research Ethics Committee (NSHD).

### Statistical analysis

Quantitative MWS variables obtained from self-reported data (current height, weight, body mass index (BMI), waist circumference, hip circumference, waist-to-hip ratio and waist-to-height ratio, birth weight, mother's and father's heights, age at menarche and age at menopause) were compared to corresponding NSHD variables in several ways. Pearson product-moment correlation coefficients were computed to investigate the strength of associations between MWS and NSHD data, and the loss of power due to reporting errors [30,31]. For each variable, possible over- or under-reporting in MWS data was assessed using the *t*-test for the difference between mean MWS and mean NSHD values. Overall agreement between corresponding MWS and NSHD variables was evaluated from the limits of agreement, computed from the means and standard deviations of the between-study differences [32]. If the NSHD data are close to the true values, the limits of agreement give the typical range of reporting errors in the MWS data. Simple error models imply that in epidemiological analyses, purely random reporting errors in exposure data cause attenuation of disease-exposure estimates, according to the ratio of the standard deviation of the errors to the standard deviation of the true values [33,34], so we interpreted the limits of agreement on the scale of the standard deviations of the NSHD values. Agreement between MWS and NSHD data was further assessed by the intraclass correlation coefficient, ICC(1,1) in the notation of Shrout and Fleiss [35]. The extent of disagreement between MWS and NSHD values, due to random or systematic errors in data from either study, is indicated by the ICC, with an ICC of 1 corresponding to perfect agreement. Substantial differences between the ICC and the Pearson correlation for a variable indicate substantial systematic differences between the MWS and NSHD data.

Systematic variation in mean over- or under-reporting across appropriate categories of the NSHD data was tested by one-way ANOVA. Systematic reporting errors may also contribute to confounding of estimated associations, which we assessed for each variable by plotting mean NSHD values against mean MWS values according to pre-specified categories of each MWS variable. Such comparisons indicate where measurements tend to be higher or lower than self-reported data would otherwise suggest. These means can be used to interpret results based on self-reported data on a more objective scale, for example by plotting the relative risks for categories of self-reported data against the mean measured values within each category. Regression dilution ratios (RDR) were calculated as the ratio of the range of the NSHD means to the range of the MWS means [36]. The use of self-reported data results in biased estimates of linear associations (e.g. log relative risks). The RDR is a non-parametric estimate of the ratio of such a biased estimate to the coefficient that would be found if analyses could be conducted using true values of the variable of interest. This ratio depends on both random error and systematic errors, but is largely independent of the true coefficient [31,37]. A regression dilution ratio close to 1 therefore indicates that there is little combined effect of random and systematic reporting errors. More often RDRs are less than 1, and provide an estimate of the relative attenuation of relative risks due to linear systematic and random reporting error. Under this assumption, estimated relative risks (RR) from univariate analyses of continuous variables can, in principle, be corrected using the RDR: the corrected relative risks would be equal to exp(ln(RR)/RDR). Confidence intervals for regression dilution ratios were obtained by bootstrapping, using the percentile method [38]. Regression dilution ratios for MWS variables are calculated under the assumption that the corresponding NSHD variables are at least "alloyed gold standard" [39] measurements of the true quantities of interest (i.e. potentially subject to a small random measurement error), and in particular that any errors in NSHD values are not correlated with other quantities of interest.

Ordinal categorical variables from the MWS (relative body size at age 10, clothes size at age 20, clothes size in middle age, bra band size in middle age) were compared with anthropometric data from the NSHD obtained at a similar age (body mass index at ages 11, 20 and 53 years, and waist and chest circumferences at age 53 years). Associations between the MWS and NSHD variables were examined using Spearman correlations between the ordinal group ranks (i.e. 1 for the lowest category, 2 for the next lowest, and so on) and the quantitative NSHD data. Linear relationships were assessed statistically by *P*-values for linear trends, and graphically by plotting means and standard errors of NSHD values against the MWS categories.

Categorical variables (having been breastfed, ever use of oral contraceptives) were compared using the raw percentage agreement and the κ statistic. The κ (kappa) statistic expresses the proportion of self-reported and measured observations which agree, over and above that which would be expected by chance [40]. A common convention is to interpret 0.2 < κ ≤ 0.4 as 'fair' agreement, 0.4 < κ ≤ 0.6 as 'moderate' agreement, 0.6 < κ ≤ 0.8 as 'substantial' agreement, and κ > 0.8 as 'almost perfect' (here referred to as 'excellent') agreement between self-reported and measured values [41].

Differences in agreement between MWS and NSHD data and proportions of women with missing values in either study were assessed for all primary variables (e.g. height and weight, but not BMI), according to childhood social class [42] and educational level in the NSHD, to adult deprivation [43] in the MWS, and to whether the participant reported that their mother was still alive at the time of the MWS resurvey, using Fisher's exact test.

## Results

There were 541 women who were participants in both studies, comprising 29% of MWS participants born in the relevant week in March 1946, and 21% of females in the original NSHD cohort. Their average age at MWS recruitment was 52 years. Of these women, 368 filled out both the recruitment and resurvey questionnaires for the MWS, with an average age at the resurvey of 55 years. Participants matched to both studies did not differ in most respects from other MWS participants born within a year of the NSHD cohort (Table 2). There was some evidence that matched participants had a small tendency to live in less deprived areas (27.3% in the most deprived tertile versus 33.1%, *P* = 0.01 for chi-squared test of association), a later mean age at menopause (48.0 years versus 47.3 years, *P* = 0.03 for ANOVA), and a very slightly earlier mean age at menarche (13.2 years versus 13.3 years, *P* = 0.02 for ANOVA).

**Table 2:** Comparison of MWS characteristics between matched participants and others of a similar age

Variable	Matched participants	MWS cohort born ± 1 yr of NSHD	*P* difference*
Number of women at baseline	541	217,828	
Number of women at resurvey	368	135,417	
Age at baseline, yr, mean	52.1	52.1	
			
Deprivation, % most deprived tertile	27.3	33.1	0.01
Maternal vital status, % alive	35.6	38.1	0.33
			
**Self-reported at recruitment in the MWS**			
Height, cm, mean (SD)	162.6 (6.4)	162.1 (6.7)	0.07
Weight, kg, mean (SD)	68.8 (13.2)	68.7 (12.9)	0.78
BMI, kg/m^2^, mean (SD)	26.0 (4.6)	26.1 (4.8)	0.35
Age at menarche, yr, mean (SD)	13.2 (1.5)	13.3 (1.6)	0.02
Age at menopause, yr, mean (SD)	48.0 (5.0)	47.3 (5.8)	0.03
Use of oral contraceptives, % ever	72.2	73.0	0.66
			
**Self-reported at resurvey in the MWS**			
Birth weight, kg, mean (SD)	3.24 (0.68)	3.22 (0.69)	0.69
Mother's height, cm, mean (SD)	160.6 (7.1)	160.4 (6.8)	0.61
Father's height, cm, mean (SD)	174.1 (7.7)	174.3 (7.8)	0.63
Waist circumference, cm, mean (SD)	75.8 (9.1)	75.7 (9.3)	0.96
Hip circumference, cm, mean (SD)	99.9 (8.1)	99.6 (7.8)	0.52
Waist-to-hip ratio, mean (SD)	0.76 (0.06)	0.76 (0.06)	0.38
Waist-to-height ratio, mean (SD)	0.47 (0.06)	0.47 (0.06)	0.99
Whether breastfed, % ever breastfed	73.0	69.7	0.23
Body size, age 10			
*% thinner*	25.8	29.9	0.23
*% average*	56.0	52.4	
*% plumper*	18.2	17.7	
Body size, age 20			
*% size < 12*	29.8	31.7	0.27
*% size 12-14*	58.1	58.4	
*% size 16+*	12.1	9.9	
Clothes size			
*% size < 14*	30.9	32.0	0.18
*% size 14-16*	44.8	49.1	
*% size 18+*	24.3	18.9	
Bra band size			
*% size 36*	22.8	23.9	0.34
*% size 36-38*	56.0	59.4	
*% size 40+*	21.3	16.7	

MWS: Million Women Study. NSHD: National Survey of Health and Development.

* *P* from ANOVA for continuous variables and from chi-squared tests of association for categorical variables.

Self-reported quantitative MWS variables showed good overall agreement with those measured in the NSHD (Table 3). For most variables, the mean between-studies difference was consistent with slight under-reporting relative to the measured quantities (*P* ≤ 0.002 for *t*-tests). Only height was significantly over-reported on average (*P* < 0.001), while age at menarche (*P* = 0.09), age at menopause (*P* = 0.09) and father's height (*P* = 0.5) showed no significant mean difference. The limits of agreement between MWS and NSHD values indicated that overall agreement was greatest for height, weight, body mass index and age at menopause. Birth weight, mother's and father's heights, waist and hip circumferences and waist-to-height ratio all had more moderate levels of overall agreement. Overall agreement was worst for age at menarche and waist-to-hip ratio. The asymmetry of limits of agreement around 0 for waist circumference, and waist-to-hip and waist-to-height ratios reflect the greater mean differences between studies for these variables. Intraclass correlations were consistent with these assessments for all variables.

**Table 3:** Comparisons between quantitative MWS and corresponding NSHD variables

				Difference		Scaled 95% limits	Intraclass	Pearson
		MWS	NSHD	(MWS - NSHD)		of agreement	correlation	correlation
				
Variable (units)	N	mean (SD)	mean (SD)	mean (SD)	P*	lower to upper†	ICC (SE) ‡	r (SE)
**Self-reported at recruitment in the MWS**								
Height (cm)	449	162.8 (6.3)	162.0 (5.7)	0.9 (2.7)	<0.001	-0.8 to 1.1	0.89 (0.01)	0.91 (0.01)
Weight (kg)	443	68.8 (13.1)	71.0 (14.1)	-2.2 (4.2)	<0.001	-0.7 to 0.4	0.94 (0.006)	0.95 (0.004)
Body mass index (kg/m^2^)	437	25.9 (4.7)	27.0 (5.2)	-1.1 (2.0)	<0.001	-1.0 to 0.5	0.90 (0.01)	0.92 (0.01)
Age at menarche (yr)	394	13.0 (1.4)	12.9 (1.0)	0.1 (1.2)	0.093	-2.1 to 2.3	0.54 (0.04)	0.57 (0.03)
Age at menopause (yr)	134	48.0 (4.5)	48.2 (4.2)	-0.3 (1.8)	0.091	-0.9 to 0.8	0.92 (0.01)	0.92 (0.01)

**Self-reported at resurvey in the MWS**								
Birth weight (kg)	234	3.2 (0.7)	3.3 (0.5)	-0.1 (0.4)	<0.001	-1.8 to 1.4	0.75 (0.03)	0.78 (0.03)
Mother's height (cm)	294	160.4 (7.1)	161.4 (6.5)	-0.9 (5.2)	0.002	-1.7 to 1.4	0.70 (0.03)	0.71 (0.03)
Father's height (cm)	240	174.1 (7.6)	173.7 (7.9)	0.3 (6.3)	0.451	-1.5 to 1.6	0.67 (0.04)	0.67 (0.04)
Waist circumference (cm)	206	76.0 (9.3)	82.0 (10.6)	-6.0 (7.2)	<0.001	-1.9 to 0.8	0.59 (0.05)	0.74 (0.03)
Hip circumference (cm)	204	100.0 (8.3)	103.1 (8.5)	-3.2 (5.3)	<0.001	-1.6 to 0.9	0.74 (0.03)	0.80 (0.03)
Waist-to-hip ratio	197	0.76 (0.06)	0.79 (0.06)	-0.03 (0.06)	<0.001	-2.4 to 1.3	0.41 (0.06)	0.52 (0.05)
Waist-to-height ratio	204	0.47 (0.06)	0.51 (0.07)	-0.04 (0.04)	<0.001	-1.9 to 0.7	0.60 (0.05)	0.75 (0.03)

MWS: Million Women Study. NSHD: National Survey of Health and Development. N: numbers of women with non-missing values for both MWS and NSHD variables. r: Pearson correlation. SE: standard error.

* *P*-value for *t*-test of mean difference = 0.

† Lower to upper Bland-Altman limits of agreement divided by the SD of the NSHD data.

‡ ICC(1,1) in the notation of Shrout and Fleiss [26].

Poorer overall agreement was typically (but not always) reflected in lower Pearson correlations between self-reported and measured variables. For two linearly related variables, the Pearson correlation coefficient measures the strength of their association, which depends on random but not systematic error. For height, weight, BMI, and age at menopause, there were very strong correlations between self-reported MWS and measured NSHD values (Pearson correlations 0.91, 0.95, 0.92 and 0.92, respectively). Self-reported birth weight, mother's height, father's height, waist circumference, hip circumference and waist-to-height ratio were also strongly correlated with NSHD data (Pearson correlations 0.78, 0.71, 0.67, 0.74, 0.80 and 0.75, respectively). MWS data on age at menarche was more moderately correlated with the NSHD values (Pearson correlation 0.57), while waist-to-hip ratio had the weakest correlation (Pearson correlation 0.52), due in part to substantial correlations between the errors in self-reported waist and hip circumferences (result not shown: Pearson correlation 0.55). Pearson correlations were somewhat larger than the intraclass correlations for waist circumference (0.74 versus 0.59), waist-to-hip ratio (0.52 versus 0.41) and waist-to-height ratio (0.75 versus 0.60), consistent with greater systematic reporting errors for these variables.

Systematic over- or under-reporting of quantitative MWS data differed, for most variables, across the distribution of NSHD values (Table 4; *P* ≤ 0.007 for one-way ANOVA). Only height (*P* = 0.6), age at menopause (*P* = 0.4) and birth weight (*P* = 0.4) showed no significant variation. Other variables were under-reported across all categories, with increased under-reporting in the highest categories of the NSHD values, except age at menarche, father's height and waist-to-hip ratio, which were each over-reported in the lowest categories and under-reported in the highest categories.

**Table 4:** Over- and under-reporting in quantitative MWS variables by quintiles of corresponding NSHD data

	1st quintile		2nd quintile		3rd quintile		4th quintile		5th quintile		
		
Variable (units)	N	mean error (SD)*	N	mean error (SD)*	N	mean error (SD)*	N	mean error (SD)*	N	mean error (SD)*	*P*†
**Self-reported at recruitment in the MWS**											
Height (cm)	91	0.6 (2.2)	93	1.0 (2.0)	86	0.8 (3.8)	89	0.7 (2.8)	90	1.1 (2.3)	0.645
Weight (kg)	92	-0.2 (3.0)	88	-1.3 (2.8)	90	-2.3 (3.8)	87	-2.6 (4.2)	86	-4.9 (5.4)	<0.001
Body mass index (kg/m^2^)	90	-0.3 (1.2)	90	-0.5 (1.8)	88	-0.9 (1.8)	87	-1.5 (1.6)	82	-2.5 (2.6)	<0.001
Age at menarche ‡ (yr)	12	1.4 (1.4)	57	0.3 (0.9)	117	0.1 (1.2)	155	0.1 (1.1)	53	-0.2 (1.2)	<0.001
Age at menopause (yr)	33	-0.1 (2.4)	26	-0.5 (2.4)	31	0.1 (1.0)	27	-0.3 (0.8)	17	-0.8 (1.3)	0.436

**Self-reported at resurvey in the MWS**											
Birth weight (kg)	61	-0.1 (0.4)	45	-0.1 (0.6)	32	0.0 (0.4)	59	-0.1 (0.4)	37	-0.2 (0.4)	0.377
Mother's height (cm)	56	-0.1 (5.9)	102	-0.2 (4.2)	52	-0.6 (4.7)	24	-1.1 (5.6)	60	-3.1 (6.0)	0.007
Father's height (cm)	47	5.1 (7.0)	70	1.5 (5.2)	51	-2.2 (5.5)	28	-2.7 (7.1)	44	-1.8 (3.7)	<0.001
Waist circumference (cm)	53	-2.6 (4.5)	43	-4.2 (6.1)	49	-6.3 (6.3)	35	-7.3 (7.2)	26	-13.8 (9.1)	<0.001
Hip circumference (cm)	50	-0.7 (4.6)	53	-3.6 (5.3)	44	-2.6 (4.7)	31	-4.2 (4.9)	26	-6.6 (6.1)	<0.001
Waist-to-hip ratio	50	0.00 (0.04)	41	-0.03 (0.04)	37	-0.03 (0.05)	39	-0.05 (0.07)	30	-0.08 (0.06)	<0.001
Waist-to-height ratio	59	-0.02 (0.03)	40	-0.03 (0.04)	45	-0.03 (0.05)	33	-0.05 (0.04)	27	-0.08 (0.06)	<0.001

MWS: Million Women Study. NSHD: National Survey of Health and Development. N: numbers of women with non-missing values for both MWS and NSHD variables. SD: standard deviation of the mean error.

* Errors calculated as the difference (MWS value - NSHD value).

† *P*-value for the F-test (ANOVA) for a difference, across quintiles of the NSHD data, in the mean error in the MWS self-reported values with respect to the NSHD values.

‡ Age at menarche categorised as <11, 11, 12, 13 or 14+ years, rather than by quintiles.

**Figure 1:**
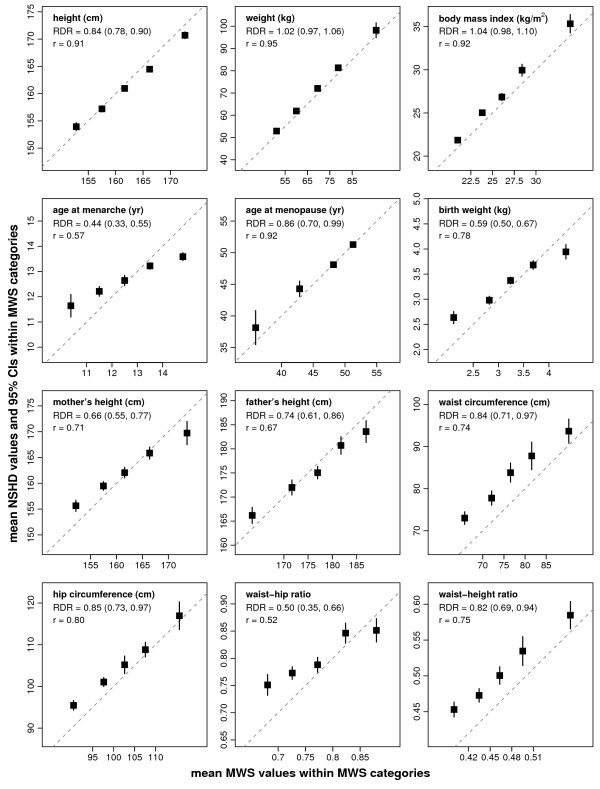
**Quantification of the effects of reporting errors in MWS anthropometry and reproductive history variables**. Means and 95% confidence intervals for NSHD variables are plotted against means of corresponding MWS variables, within selected categories of the MWS data. Category boundaries are given on the horizontal axes. Pearson correlation coefficients (r) are indicated for each variable, as are regression dilution ratios (RDR) with 95% bootstrapped confidence bounds, indicating the likely relative attenuation of linear coefficients for disease-exposure associations. The dashed lines are lines of equality of means.

For most quantitative variables, categories of self-reported MWS data were characterised by distinct means of the corresponding measured values (Figure 1). Values above or below the dashed line of equality in the figure indicate where NSHD values are typically larger or smaller, respectively, than the self-reported data would suggest. For waist-to-hip ratio, however, there was little relationship between reported and measured values in the two upper categories of the MWS data. Regression dilution ratios broadly reflected correlations between the two studies. The RDRs for weight (RDR = 1.02, 95% CI 0.97-1.06) and body mass index (1.04, 0.98-1.10) indicate that for these variables, differential systematic errors effectively cancel out the likely attenuation of risk estimates due to random errors. Most other quantitative variables had RDRs in the range 0.66-0.86, consistent with slight to moderate attenuation, with the exceptions of age at menarche (RDR = 0.44, 0.33-0.55), birth weight (0.59, 0.50-0.67) and waist-to-hip ratio (0.50, 0.35-0.66), for which more substantial attenuation is likely.

**Figure 2:**
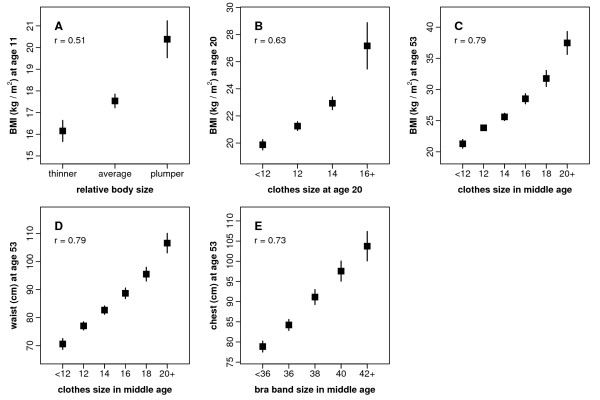
**Comparisons of NSHD anthropometry against corresponding ordinal MWS body size variables at various ages**. Means and 95% confidence intervals of NSHD anthropometric variables are plotted according to categories of MWS body size variables. (A) NSHD BMI measured at age 11 versus MWS relative body size at age 10; (B) NSHD BMI reported at age 20 versus MWS clothes size at age 20; (C) NSHD BMI measured at age 53 versus MWS clothes size in middle age; (D) NSHD waist circumference measured at age 53 versus MWS clothes size in middle age; (E) NSHD chest circumference measured at age 53 versus MWS bra band size in middle age. Spearman correlations (r) are indicated for each pair of NSHD and MWS variables.

Ordinal MWS body size variables reported at resurvey (relative body size at age 10; clothes size at age 20; clothes size at resurvey; bra band size at resurvey) show clear associations with anthropometry recorded at the corresponding ages by the NSHD (Figure 2). The closer the ages at which data were collected by the NSHD and MWS, the stronger the associations (Table 5). Correlations were moderate for relative body size at age 10 (Spearman correlation, 0.51) and clothes size at age 20 (Spearman correlation 0.63), and strong for current clothes size compared either with BMI at age 53 (Spearman correlation 0.79) or with waist circumference at age 53 (Spearman correlation 0.79), and for current bra band size (Spearman correlation 0.73). There were significant trends in measured anthropometry across categories of all self-reported ordinal body size variables (*P* < 0.001).

**Table 5:** Associations between ordinal MWS body size variables at various ages and corresponding NSHD anthropometry

			Spearman	*P*
MWS variable and age	NSHD variable (units) and age	N	r*	(trend)†
Relative body size at age 10	Body mass index (kg/m^2^) at age 11	306	0.51	<0.001
Clothes size at age 20	Body mass index (kg/m^2^) at age 20	302	0.63	<0.001
Clothes size at resurvey	Body mass index (kg/m^2^) at age 53	316	0.79	<0.001
Clothes size at resurvey	Waist circumference (cm) at age 53	316	0.79	<0.001
Bra band size at resurvey	Chest circumference (cm) at age 53	291	0.73	<0.001

MWS: Million Women Study. NSHD: National Survey of Health and Development. N: numbers of women with non-missing values for both the MWS and NSHD variables.

* Spearman rank correlation coefficient for the strength of a monotonic relationship between the ordinal MWS variable and the continuous NSHD variable.

† *P*-values for overall significance of linear regressions of continuous NSHD values against ordinal MWS values.

Categorical data on factors related to reproductive history showed moderate to excellent agreement with information recorded in the NSHD. Report at MWS recruitment of past use of oral contraceptives had an excellent level of agreement with ever use of oral contraceptives obtained by combining NSHD data collected at ages 31 and 43 years (κ = 0.87, 94.8% agreement; 482 women with non-missing data). MWS and NSHD data on whether the women were ever breastfed (yes/no data in the MWS corresponding in the NSHD to the mother's report of breastfeeding for even a short time) had a high percentage agreement (81.0%; 268 women), but only moderate agreement according to the κ statistic (κ = 0.48). Agreement was higher between the MWS data (yes/no) and report in the NSHD that the woman was breastfed for at least 1 month rather than never having been breastfed or breastfeeding having stopped within the first month (κ = 0.58, 82.8% agreement). Agreement was significantly greater than that expected by chance for both variables (*P* < 0.001).

There were few significant variations in mean difference or agreement across categories of childhood social class, educational attainment, adult deprivation or whether the participant's mother was still alive at MWS resurvey (*P* > 0.05 for chi-squared test of association). The only exceptions were age at menarche, which varied according to tertiles of adult deprivation (*P* = 0.02) but with no particular trend, and mother's height, for which there was greater under-reporting by participants with living mothers (*P* = 0.02). There were, however, significant differences (*P* < 0.05) in the proportion of missing data according to childhood social class (for height, weight, waist and hip circumferences, and ever use of oral contraceptives), according to educational attainment (for height, weight, waist and hip circumferences, clothes size at age 20, and ever use of oral contraceptives), according to adult deprivation (for height and weight) and according to whether the participant's mother was still alive (for weight and birth weight, and whether the participant was breastfed). For all variables, individuals were more likely to have missing data if they had a lower childhood social class, greater adult deprivation, lower educational attainment or if their mother was no longer alive.

## Discussion

The present paper is one of the few to attempt to directly quantify the likely effects of reporting error on disease-exposure associations for any anthropometric or reproductive history variables [23-27]. Purely random errors in reported values bias estimates towards the null, but inflation of estimates is also possible if systematic reporting errors work in opposition to the effects of random errors, or where errors in adjustment factors are correlated with those in the main exposure [23,33]. For epidemiological analyses, the utility of self-reported exposure data is determined by the magnitudes of these errors, the attendant loss of power, and whether biases in estimates can be corrected either formally or informally. Methods of correction for random and systematic measurement or reporting errors, such as the regression calibration methods of Rosner et al. [44] and later developments thereof, have been used extensively in nutritional epidemiology, where discrepancies between reported and true dietary intakes can be substantial [33,45], but in few other areas of epidemiology. The regression dilution ratio approach was developed in the context of prospective studies of clinical measurements such as blood pressure [36], which has relatively poor repeatability over time. Regression dilution ratios estimate the same quantity as the regression calibration methods familiar to nutritional epidemiologists [23], and can be applied, as we have done, to general measurement or reporting error problems in non-clinical contexts.

In contrast to statistics for agreement, which are purely descriptive, regression dilution ratios summarise the potential consequences of both random and systematic errors for epidemiological analyses. We found RDRs consistent with slight to moderate attenuation of estimates of disease-exposure associations (RDRs 0.66-0.86) for most quantitative anthropometric and reproductive history variables. A few variables (age at menarche, birth weight and waist-to-hip ratio) had smaller RDRs, consistent with more substantial attenuation of estimates (RDRs 0.44-0.50). For weight (RDR 1.02) and body mass index (RDR 1.04), however, there was little attenuation.

These regression dilution ratios provide a guide to possible effects of reporting error in one particular cohort, although in principle a good estimate of the regression dilution ratio can be used to correct estimates of linear disease-exposure associations in univariate analyses. For example, a regression dilution ratio of 0.5 corresponds to a 50% attenuation of the log relative risk (or other linear coefficient) towards 0. An estimated relative risk of 1.5 per unit self-reported exposure would then, after correction for reporting error, be equal to exp(ln(1.5)/0.5) = 2.25 per unit true exposure.

Regression dilution ratios are not suitable for correcting estimates of non-linear disease-exposure associations, such as the apparent J-shape in the association between BMI and all-cause mortality [27]. In these cases, the means presented in Figure 1 provide a guide to a more objective scale on which to interpret relative risks across categories of these variables. For example, relative risks within categories of BMI or other variables could be plotted against mean measured values. In addition, regression dilution ratios will not reveal situations where self-reported values are not linearly related to the reference values. However, the approximate linearity of each plot in Figure 1 (with the possible exception of the plot for waist-to-hip ratio) indicates that RDRs will provide suitable summaries of the effects of reporting errors across the ranges of each of these variables. Regression dilution ratios and the mean reference values presented in the figures are calculated under the additional assumption that NSHD reference values are unbiased but may be subject to small random errors that are uncorrelated with other quantities of interest. Results for regression calibration methods suggest that even if these assumptions are violated, imperfect adjustment for reporting error is usually better than proceeding with analyses under the false presumption that exposures are self-reported without error [39].

It must also be emphasised that methods of correction for reporting error, including the use of regression dilution ratios, are not robust to other common statistical problems. Poorly assessed outcomes, violations of assumptions underlying statistical methods, and lack of information on confounders, among other issues, can result in bias to estimates which will remain even after accounting for reporting error.

Systematic and random reporting errors also result in a loss of power to correctly reject false null hypotheses of no effect. Squared correlation coefficients indicate the approximate effective sample sizes, as a proportion of actual sample sizes, due to loss of power [30,31]. Correlations reported here are consistent with reductions in effective sample sizes of between 9%, for weight, and 73%, for waist-to-hip ratio. Importantly, loss of power due to reporting errors cannot be remedied by correcting estimates using RDRs or similar techniques. The sample size must also be increased, and consequently regression dilution ratios and other methods for accounting for bias due to reporting error will be most useful in large-scale studies, or those that are otherwise well-powered. (Sample size calculations for studies based on self-reported data will still be accurate, however, provided that they are interpreted as sample sizes required to detect the attenuated association between the disease and the self-reported exposure.)

We also found good overall agreement between MWS and NSHD data for quantitative anthropometric and reproductive history variables, particularly for current height, weight and body mass index reported at recruitment. However, consistent with findings of previous studies [2,11,12,15,16], differences between MWS and NSHD anthropometric data included systematic over-reporting of height, and under-reporting of weight that was more pronounced among heavier individuals. Similar differential under-reporting was observed for self-reported waist and hip circumferences [6,13], recalled body size variables including childhood body size and birth weight [4,7-10,18], and reported body sizes of close relatives [5]. Comparisons between intraclass and Pearson correlations suggested that systematic reporting errors were relatively greater for waist circumference and for the derived waist-to-hip and waist-to-height ratios, than they were for other variables. For both weight and body mass index, the increased under-reporting among heavier individuals explains why their regression dilution ratios are close to 1: this differential under-reporting would inflate estimates of disease-exposure associations, counteracting the attenuation due to random reporting errors. The RDRs for other variables (except height, birth weight, and age at menarche) may also be closer to 1 than would result from random error alone, due to increased under-reporting of each variable in its upper range of values. Differential under-reporting also implies that self-reported anthropometric data are likely to be inadequate for the purposes of clinical assessment, for example when classifying an individual as normal weight, overweight or obese based on their body mass index.

Most MWS variables on reproductive history and related factors showed good to moderate agreement with NSHD data. The exception was age at menarche, for which there was poorer agreement between the MWS and NSHD data. This level of agreement was comparable to that found in a recent validation study of recalled age at menarche in a larger subset of NSHD participants, which concluded that age at menarche self-reported in middle age may not be appropriate in a clinical setting, or to estimate risk profiles for associated diseases [22]. Several previous studies have concluded that information on having been breastfed, age at menopause and use of oral contraceptives is recalled with reasonable accuracy [17-21], however it is generally advisable to be cautious in the use of data that is recalled many years after the time of interest [22].

We also compared ordinal body size variables from the MWS, self-reported in middle age (relative body size at age 10, clothes size at age 20 and at recruitment and bra band size at recruitment), with anthropometry from the NSHD collected at the relevant ages (body mass index, waist circumference and chest circumference). Ordinal body size variables from the MWS were moderately to strongly associated with the NSHD variables against which they were compared. Notably, the strength of the relationship between clothes size reported at resurvey and measured waist circumference was comparable to that between reported waist circumference and measured waist circumference. This suggests that for the purposes of epidemiological studies, self-reported clothes size might be at least as good a proxy for waist measurements as self-reported waist circumference. Other studies have found differential systematic error in reported anthropometry in childhood and early adulthood (e.g. again, greater under-reporting of weight by heavier individuals) [3,4,10]. For ordinal data, however, it is not possible to assess agreement with anthropometry. Our results focus instead on the strength of the association between ordinal variables and corresponding anthropometry.

We are unaware of any studies which have directly validated self-reported clothes sizes against actual clothes sizes in either men or women, but in men measured trouser-waist size has been found to be highly correlated (r > 0.85) with clinical measurement of waist circumference [46]. Our findings suggest that clothes size might be well-reported by women and be representative of their true body size. Few studies have used clothes sizes as markers of disease risk [14,46,47], but the relationships they find are consistent with those for more conventional anthropometry. The mean NSHD values presented by category of clothes size and other ordinal variables (Figure 2) can be used in the interpretation of these relationships on a more objective scale.

Although most variables were validated against measured values or information from other reliable sources, clothes size at age 20 and maternal height were validated against data that was self-reported at the relevant age, and father's height and age at menarche were validated against data reported by proxy. In these cases, despite being collected close to the relevant time the reference NSHD data are not "gold standard". Hence there are two major sources of error: first, in the self-reported or proxy NSHD data, and second, in the self-reported MWS data. Because our results for these variables can at most account only for the second source of error, it is likely that they overestimate, to some degree, the levels of association and agreement between the two studies. Similarly, regression dilution ratios for MWS data on parental heights may underestimate the effects of error in these variables, which is likely to result in greater attenuation of estimates in epidemiological studies.

Other types of error are included within reporting error, but should be considered when interpreting any statistics for association and agreement, and regression dilution ratios. Survey questions were developed independently for each study. For data that was self-reported in both studies, subtle differences in wording of questions, and differences in the requested precision of responses, could contribute to disagreement between the studies. There were also variations in differences between the age at which NSHD data were collected and the age of data collection or referent age for MWS data (e.g. a difference between studies of 2.3 years between the average age of collection of waist and hip measures). These differences may contribute to slightly greater apparent reporting error for some variables than would have been found if the ages could have been matched more closely. Conversely, reporting errors assessed here do not include changes in exposures during follow-up, such as has been observed for blood pressure [24,36] and may be likely for anthropometric variables including weight. Prospective studies with a long period of follow-up should also assess the contribution of such changes over time to bias in disease-exposure associations [24].

There were few significant associations of reporting errors in the variables considered in this study with childhood social class, educational attainment, adult deprivation and whether the participant's mother was still alive. However, there were more missing values in the lower socio-economic groups, and comparisons may not be generalisable to all subgroups of these factors. Overall comparisons between variables and detailed assessments of between-study differences by socio-economic group may be further limited by small numbers, particularly for age at menopause and variables reported at MWS resurvey. One other study has found no association of between-study differences in body weight according to socio-economic factors [3], but several studies have found differences in reporting of anthropometry according to sex, age, education or ethnicity [1,16,48,49]. Other than education, we were unable to assess these factors, due to the composition of the cohort. Further investigations of populations including men, or with different distributions of ages, socio-economic factors or ethnicities, will be required to determine whether regression dilution ratios are similar, in these other populations, to the results presented here.

A previous report from the NSHD showed that categorical agreement between age at menarche reported during adulthood and that recorded nearer the time can vary according educational attainment [22]. Similar to the other variables, we found no significant associations of quantitative between-study differences with childhood social class or educational attainment for age at menarche. Because age at menarche was reported by proxy, the magnitude and effects of reporting errors could be underestimated, though it seems likely that a participant's mother would have been able to report her daughter's age at menarche with reasonable accuracy, at the time she was asked. Also, quantitative NSHD data on age at menarche is limited to women with age of menarche at most 14-15 years. This limitation could result in exaggerated between-studies differences for women reporting older ages at menarche in the MWS. For age at menopause reported at recruitment, because women matched to both studies were at most 55 years old when they joined the MWS, it was not possible to compare MWS ages at menopause greater than 55 years against NSHD data. Agreement between the studies for age at menopause was very high, although this may in part be due to improved recall in the MWS as a result of the very frequent follow-up for age at menopause, in the NSHD, between the ages of 47 and 54.

The matched participants in this validation study have consented to be part of two prospective cohorts, which suggests potential for self-selection biases in their data. There were few differences, however, in means of quantitative variables or proportions of categorical data between the matched participants and other MWS participants born within 1 year of the NSHD recruitment period, consistent with little additional bias. Nonetheless, the NSHD cohort has been followed since birth and participants are accustomed to providing information about their health and lifestyle, and might therefore be better able to recall information about past health and lifestyle than other women.

## Conclusions

Most of the self-reported Million Women Study data we examined showed moderate to good overall agreement with corresponding data measured or collected close to the relevant time in the MRC National Survey of Health and Development. However, reporting errors in MWS data relative to NSHD data showed both random and systematic components, consistent with those found in other studies. Although these reporting errors can be problematic for clinical interpretations of data, we focussed on the likely effects of these errors on estimates of disease-exposure associations for epidemiological studies. In this context, regression dilution ratios (or related methods) can be used as a guide to the likely attenuation of linear relative risk estimates. Mean measured values within categories of self-reported data can be used in the interpretation of relative risks across categories of either continuous or ordinal data, in those cases where disease-exposure associations might be non-linear. Regression dilution ratios for most MWS lifetime body size and reproductive history variables were consistent with slight to moderate attenuation due to reporting errors. If estimates of the effects of reporting errors are used to guide interpretation of study results, these self-reported data may be adequate for use in large epidemiological analyses. Nonetheless, larger validation studies with greater variations in age, ethnicity and other participant characteristics are needed to establish whether the results of the present study are more widely applicable. Indeed, examination of random and systematic reporting errors and their effects on estimates of disease-exposure associations should be routine in all studies that are based on self-reported exposure data.

## Competing interests

The authors declare that they have no competing interests.

## Authors' contributions

All authors were involved in designing the study and reviewed and revised drafts of the manuscript, and read and approved the final manuscript. BC devised and conducted the analyses and drafted the manuscript. BL and SC coordinated the linkage of the datasets. RC and GR assisted in planning the analyses. VB and DK are the principal investigators for the Million Women Study and MRC National Survey of Health and Development, respectively, and are responsible for data collection and study management.

## Pre-publication history

The pre-publication history for this paper can be accessed here:

http://www.biomedcentral.com/1471-2288/11/7/prepub
